# Prevalence and Characterization of Monophasic *Salmonella* Serovar 1,4,[5],12:i:- of Food Origin in China

**DOI:** 10.1371/journal.pone.0137967

**Published:** 2015-09-11

**Authors:** Xiaojuan Yang, Qingping Wu, Jumei Zhang, Jiahui Huang, Weipeng Guo, Shuzhen Cai

**Affiliations:** 1 Guangdong Institute of Microbiology, State Key Laboratory of Applied Microbiology Southern China, Guangzhou, China; 2 Guangdong Provincial Key Laboratory of Microbial Culture Collection and Application, Guangdong Open Laboratory of Applied Microbiology, Guangzhou, China; Panjab University, INDIA

## Abstract

*Salmonella enterica* subsp. *enterica* serovar 1,4,[5],12:i:- is a monophasic variant of *Salmonella* Typhimurium, which has recently been recognized as an emerging cause of infection worldwide. This bacterium has also ranked among the four most frequent serovars causing human salmonellosis in China. However, there are no reports on its contamination in Chinese food. Serotyping, polymerase chain reaction, antibiotic resistance, virulotyping, and multilocus sequence typing (MLST) assays were used to investigate the prevalence of this serological variant in food products in China, and to determine phenotypic and genotypic difference of monophasic isolates and *Salmonella* Typhimurium isolated over the same period. *Salmonella* 1,4,[5],12:i:- was prevalent in various food sources, including beef, pork, chicken, and pigeon. The study also confirmed the high prevalence (53.8%) of resistance to ampicillin, streptomycin, sulfonamides, and tetracycline in *Salmonella* 1,4,[5],12:i:-, which was higher than that in *Salmonella* Typhimurium. Moreover, *Salmonella* 1,4,[5],12:i:- isolates in our study were different from *Salmonella* Typhimurium isolates by the absence of three plasmid-borne genes (*spvC*, *pefA*, and *rck*) and the presence of *gipA* in all isolates. All *Salmonella* 1,4,[5],12:i:- isolates demonstrated MLST pattern ST34. Genomic deletions within the *fljBA* operon and surrounding genes were only found in *Salmonella* 1,4,[5],12:i:- isolates, with all isolates containing a deletion of *fljB*. However, *hin* and *iroB* were identified in all *Salmonella* 1,4,[5],12:i:- isolates. Three different deletion profiles were observed and two of them were different from the reported *Salmonella* 1,4,[5],12:i:- clones from Spain, America, and Italy, which provided some new evidence on the independent evolution of the multiple successful monophasic clones from *Salmonella* Typhimurium ancestors. This study is the first report of *Salmonella* 1,4,[5],12:i:- in food products from China. The data are more comprehensive and representative, providing valuable information for epidemiological studies, risk management, and public health strategies.

## Introduction

*Salmonella* are often acquired from contaminated food, and are important causes of gastroenteritis and bacteremia, posing a worldwide threat to public health [1]. There are estimated to be 94 million cases of gastroenteritis globally per year, with 155,000 deaths attributed to *Salmonella* [2,3].

Since 2009, *Salmonella enterica* subsp. *enterica* serovar 1,4,[5],12:i:- has ranked among the four most frequent serovars causing human salmonellosis in China [2]. It is also one of the most common serovars isolated from humans and foods in several other countries [4,5]. This atypical serovar, lacking the phase 2 flagellar antigen [6,7], has become increasingly important since the mid-1990s worldwide. Currently, it is designated as a monophasic variant of *Salmonella* Typhimurium, with antigenic and genotypic similarities [6,8–10].

The mechanisms for the evolution of *Salmonella* 1,4,[5],12:i:- remain unknown. Several genes, including *fljB*, *fljA*, and *hin*, located within the *fljBA* operon, are involved in expression of phase 2 flagellar antigen. *fljB* encodes the phase 2 flagellar antigen, while *fljA* (encoding a negative regulator of *fliC*, coding for the phase 1 flagellar antigen) and *hin* (encoding a DNA invertase) are responsible for flagellar phase variation, a regulatory mechanism involving switching between the production of FliC or FljB [5]. Thus, mutations, deletions, or insertions of *fljB* itself, along with disorder in the regulatory mechanism of phase variation, could result in failure to express the phase 2 flagellar antigen [7,10–11]. Interestingly, various *fljB* deletion profiles, as well as deletions within other genes of the *fljBA* operon and surrounding genes (*iroB* and STM2757), have been detected [10,12–14].

Moreover, the public health impact of *Salmonella* 1,4,[5],12:i:- infection is increased by the high rate of antimicrobial resistance associated with this serovar. In particular, an epidemic multidrug-resistant (MDR) clonal lineage of *Salmonella* 1,4,[5],12:i:-, designated the European clone, which is resistant to ampicillin, streptomycin, sulfonamides, and tetracycline (R-type ASSuT) [15], has emerged in Europe and has been implicated in several outbreaks [16–18]. Some *Salmonella* 1,4,[5],12:i:- isolates from Spain, America, and Canada with additional resistance to ampicillin, chloramphenicol, streptomycin, sulfonamides, and tetracycline (R-type ACSSuT) [6,19,20] have also been detected.

Several previous studies have examined *Salmonella* 1,4,[5],12:i:- from human cases of food poisoning in China [2,21]; however, systematic data for the prevalence of *Salmonella* 1,4,[5],12:i:- in the food chain is lacking. Therefore, the objective of this study was to characterize *Salmonella* 1,4,[5],12:i:- isolates collected from food samples across China from 2011–2014, and compare these isolates with *Salmonella* Typhimurium isolated in the same period to better understand the prevalence, phenotypic and genetic diversity, and susceptibility to antimicrobials of this serovar in China. Such data will provide a scientific basis for the development of food safety regulations.

## Materials and Methods

### Bacterial strains

From July 2011 to May 2014, 2800 food samples were collected from retail markets; these included meat and meat products (639), aquatic products (554), quick-frozen food (316), edible mushrooms (270), vegetables (272), dairy products (186), fruit (10), and ready-to-eat food (553). The sampling sites were distributed throughout the 24 provincial capitals of China (S1 Fig). In total, 524 *Salmonella* isolates were obtained and identified to serovar level according to the Kauffmann-White scheme [22]. For monophasic *Salmonella* serovar 1,4,[5],12:i:-, the absence of phase 2 flagellar antigen was verified using the flagellar phase reversal method as described by Chiou et al [23]. Strains were definitively assigned to *Salmonella* Typhimurium or *Salmonella* 1,4,[5],12:i:- using the duplex-PCR assay developed by Tennant et al [24] and recommended by the European Food Safety Authority (EFSA) Panel on Biological Hazards [4]. The malic acid dehydrogenase gene (*mdh*), which is specific to *Salmonella* Typhimurium and *Salmonella* 1,4,[5],12:i:-, was also detected using primers previously described [8]. Ratios of *Salmonella* 1,4,[5],12:i:- occurrence were calculated against all serotyped *Salmonella* or *Salmonella* Typhimurium isolated from the same batch of food samples in the same period [11].

### Antimicrobial susceptibility testing

Antimicrobial susceptibility was evaluated using the Kirby-Bauer disk diffusion method with 20 antimicrobial agents, in accordance with the Clinical Laboratory Standards Institute guidelines [25]. The antimicrobials used were ampicillin, amoxicillin-clavulanic acid, cephalothin, cefazolin, cefoxitin, ceftriaxone, cefotaxime, ceftazidime, cefoperazone, cefepime, chloramphenicol, tetracycline, nalidixic acid, ciprofloxacin, amikacin, gentamicin, streptomycin, kanamycin, trimethoprim-sulfamethoxazole, and sulfonamides (Oxoid, Basingstoke, UK).

### Detection of virulence-related genes

Seven genes with reported contributions to virulence were selected. Four targets (*gipA*, *sodC1*, *sopE1*, *sspH1*) were located on prophages, while three (*spvC*, *pefA*, *rck*) were located on a virulence plasmid. Amplification was performed using primers and conditions described previously [26,27]. *Salmonella* Typhimurium ATCC14028 and *Salmonella* Typhimurium CMCC50115 were used as positive controls.

### Multilocus sequence typing (MLST)

*Salmonella* isolates were characterized by MLST using previously reported primers specific for seven housekeeping genes: *aroC*, *dnaN*, *hemD*, *hisD*, *purE*, *sucA*, and *thrA*. The sequence type (ST) of each isolate was assigned according to the MLST database (http://mlst.ucc.ie/mlst).

### PCR-based characterization of gene deletion profiles of *Salmonella* isolates

For all *Salmonella* Typhimurium and *Salmonella* 1,4,[5],12:i:- isolates, the presence of *fljA*, *fljB*, and *hin*, as well as *iroB*, which flanks the *fljBA* operon, and the closely located STM2757 and STM2758 genes, were analyzed using gene-specific primers and PCR conditions as previously described by Soyer et al [10] and García et al [12]. *fljA*, *fljB*, *hin*, and *iroB* are located at the 3′ end of cluster V, while STM2757 and STM2758 flank the 5′ end. This region spans 16 genes (from STM2758 to *iroB*) that were previously reported to be present in *Salmonella* Typhimurium LT2, but absent in a selection of Spanish *Salmonella* 1,4,[5],12:i:- isolates [28].

## Results

As shown in S1 Table and S2 Fig, serotyping, including flagellar phase reversal, identified 13 *Salmonella* isolates lacking phase 2 flagellar antigen. These *Salmonella* 1,4,[5],12:i:- isolates originated from beef (n = 6), pork (n = 5), chicken (n = 1), and pigeon (n = 1). All isolates produced a single 1,000-bp amplicon corresponding to a fragment of the IS200 element, typical of *Salmonella* 1,4,[5],12:i:-. The control group of 58 *Salmonella* Typhimurium isolates were also confirmed, with 55 isolates producing two amplicons (1,000-bp and 1,389-bp), which is specific for *Salmonella* Typhimurium, and the remaining three being positive for the 1,389-bp phase 2 flagellar gene but negative for the 1,000-bp IS200 fragment. Positive detection of *mdh* confirms the presence of *Salmonella* Typhimurium or its monophasic variant *Salmonella* 1,4,[5],12:i:-. In the current study, *mdh* was detected in all *Salmonella* 1,4,[5],12:i:- and *Salmonella* Typhimurium isolates. The overall *Salmonella* 1,4,[5],12:i:- occurrence ratio reached 2.5% (13/524), whereas *Salmonella* 1,4,[5],12:i:-/*Salmonella* Typhimurium ratio was 22.4% (13/58) in the period of 2011 to 2014.

All 71 of the *Salmonella* isolates examined in this study showed some level of antibiotic resistance. In total, 76.9% of *Salmonella* 1,4,[5],12:i:- and 58.6% of *Salmonella* Typhimurium isolates were resistant to three or more antimicrobials (Table 1). Among these antibiotics, high rates of resistance were observed for tetracycline, sulfonamides, ampicillin, nalidixic acid, streptomycin, kanamycin, trimethoprim-sulfamethoxazole, and chloramphenicol in both *Salmonella* 1,4,[5],12:i:- and *Salmonella* Typhimurium isolates (S2 and S3 Tables). The main antimicrobial resistance patterns of the 13 *Salmonella* 1,4,[5],12:i:- and 58 *Salmonella* Typhimurium isolates are shown in Table 1.

**Table 1:** Antimicrobial resistance patterns of *Salmonella* 1,4,[5],12:i:- and *Salmonella* Typhimurium isolates.

R-types	*Salmonella* 1,4,[5],12:i:-,n (%)	*Salmonella* Typhimurium,n (%)
**ASSuT** ^a^ **±other**	7 (53.8%)	13 (22.4%)
**ACSSuT** ^b^ **±other**	3 (23.1%)	7 (12.1%)
**Pansusceptible**	0 (0.0%)	0 (0.0%)
**≥1 Antimicrobial**	13 (100.0%)	56 (96.6%)
**≥3 Antimicrobial**	10 (76.9%)	34 (58.6%)
**Total**	13 (100.0%)	58 (100.0%)

^a^ASSuT resistance phenotype (ampicillin-streptomycin-sulfonamides-tetracycline);

^b^ACSSuT resistance phenotype (ampicillin-chloramphenicol-streptomycin-sulfonamides-tetracycline).

The 13 *Salmonella* 1,4,[5],12:i:- isolates showed highly similar virulence gene profiles. All isolates contained *gipA* (encoding a Peyer’s patch-specific virulence factor) and *sodC1* (putative Cu/Zn superoxide dismutase), carried by the Gifsy-1 and Gifsy-2 lambdoid prophages, respectively, while *sspH1* (encoding a *Salmonella* type III effector protein), located in Gifsy-3, and three genes associated with the *Salmonella* virulence plasmid (*spvC*, *pefA*, and *rck*) were absent. However, three *Salmonella* 1,4,[5],12:i:- isolates shared a different virulence gene profile, and were positive for *sopE1* (*Salmonella* type III effector protein). Overall, the prevalence of each of the virulence genes was similar between the *Salmonella* 1,4,[5],12:i:- and *Salmonella* Typhimurium isolates, except for the absence of the three plasmid genes and presence of *gipA* in all of the *Salmonella* 1,4,[5],12:i:- isolates (Tables 2 and 3).

**Table 2:** Virulotyping: most commonly detected haplotypes.

Serotypes	Haplotypes	Number of strains
***Salmonella*** **1** **,4,[5],12:i:-**		
	*gipA*; *sodC1* ^a^	n = 10 (ST34)
	*gipA*; *sodC1*; *sopE1*	n = 3 (ST34)
***Salmonella* Typhimurium**		
	*gipA*; *sodC1*; *spvC*; *pefA*; *rck*	n = 33 (ST19); n = 2 (ST1544); n = 1 (ST1922)
	*gipA*; *sodC1* ^a^	n = 10 (ST34); n = 2 (ST19)
	*sodC1*	n = 7 (ST19)
	*sodC1*; *spvC*; *pefA*; *rck*	n = 2 (ST19)
	*sodC1*; *sopE1*	n = 1 (ST36)

^a^Identical haplotypes.

**Table 3:** Prevalence (%) of the virulence genes by serotype.

Serotypes	Gene targets						
	*gipA*	*sodC1*	*sopE1*	*sspH1*	*spvC*	*pefA*	*rck*
***Salmonella*** **1** **,4,[5],12:i:-**	100.0%	100.0%	23.1%	0.0%	0.0%	0.0%	0.0%
***Salmonella* Typhimurium**	82.8%	100.0%	1.7%	0.0%	65.5%	65.5%	65.5%

Among the 13 *Salmonella* 1,4,[5],12:i:- and 58 *Salmonella* Typhimurium isolates, five distinct STs were identified based on a seven-gene MLST scheme (Table 4). The number of alleles for the seven housekeeping genes ranged from 2–554. *hisD*554 and ST1922 were identified as a novel allele type and a novel MLST pattern, respectively. Two STs (ST19 and ST34) were predominant, and together accounted for 94.4% of isolates tested. ST19 was the most common *Salmonella* Typhimurium ST, accounting for 44 of 58 isolates. All the *Salmonella* 1,4,[5],12:i:- isolates were assigned to ST34, a single-locus variant of ST19 (with *dnaN*19 instead of *dnaN*7); 10 *Salmonella* Typhimurium isolates were also designated ST34. One *Salmonella* Typhimurium isolate was identified as ST36, with two further isolates assigned to ST1544. These three isolates also differed from other *Salmonella* Typhimurium isolates based on duplex-PCR results (positive for the 1,389-bp phase 2 flagellar gene, but negative for the 1,000-bp IS200 fragment).

**Table 4:** Allelic profiles and MLST types of the 71 *Salmonella* isolates.

Serotypes (no. of isolates)	Allelic types							MLST patterns
	*aroC*	*dnaN*	*hemD*	*hisD*	*purE*	*sucA*	*thrA*	
*Salmonella* Typhimurium (44)	10	7	12	9	5	9	2	ST19
*Salmonella* Typhimurium (10); *Salmonella* 1,4,[5],12:i:- (13)	10	19	12	9	5	9	2	ST34
*Salmonella* Typhimurium (1)	10	7	12	554*	5	9	2	ST1922*^a^
*Salmonella* Typhimurium (2)	10	7	12	230	5	9	2	ST1544
*Salmonella* Typhimurium (1)	18	14	12	9	5	18	21	ST36

^a^Asterisks denote novel alleles and novel MLST patterns.

Genomic deletions within the *fljBA* operon were only found in *Salmonella* 1,4,[5],12:i:- isolates. The 13 *Salmonella* 1,4,[5],12:i:- isolates showed three different deletion profiles for cluster V, namely *ΔfljAB*-I, *ΔfljAB*-II, and *ΔfljAB*-III. *ΔfljAB*-I and *ΔfljAB*-II were the most common deletion profiles. *fljB* was absent and *hin* was present in all *Salmonella* 1,4,[5],12:i:- isolates. However, *fljA* was present in only one isolate (deletion profile *ΔfljAB*-III). The deletion profiles *ΔfljAB*-I and *ΔfljAB*-II were marked by the presence and absence of STM2757 and STM2758, respectively (Table 5 and S3 Fig).

**Table 5:** Gene deletion profiles and types for the 13 *Salmonella* 1,4,[5],12:i:- isolates.

Isolates	Target genes						Detection types
	*hin*	*fljB*	*fljA*	*iroB*	STM2758	STM2757	
2011–33	+	-	-	+	+	+	*ΔfljAB*-I
2011–34	+	-	-	+	+	+	*ΔfljAB*-I
2012–35	+	-	-	+	-	-	*ΔfljAB*-II
2012–36	+	-	-	+	+	+	*ΔfljAB*-I
2012–37	+	-	-	+	+	+	*ΔfljAB*-I
2013–45	+	-	-	+	-	-	*ΔfljAB*-II
2013–50	+	-	-	+	+	+	*ΔfljAB*-I
2013–51	+	-	+	+	+	+	*ΔfljAB*-III
2013–52	+	-	-	+	+	+	*ΔfljAB*-I
2014–54	+	-	-	+	-	-	*ΔfljAB*-II
2013–57	+	-	-	+	-	-	*ΔfljAB*-II
2013–58	+	-	-	+	-	-	*ΔfljAB*-II
2013–62	+	-	-	+	-	-	*ΔfljAB*-II

## Discussion

This study confirmed the presence of *Salmonella* 1,4,[5],12:i:- in food products in China. The *Salmonella* isolates examined in this study were collected from a nationwide food investigation covering most provincial capitals of China. Thus, the results were fairly comprehensive and representative of China as a whole. The occurrence of *Salmonella* 1,4,[5],12:i:- was higher in our study compared with other countries [11,29].

Previous studies have suggested that the vast majority of these monophasic *Salmonella* strains are from pigs and pork products [30,31], followed by isolates obtained from poultry and cattle. Isolation from other sources was believed to be rare [9,32]. In our study, although *Salmonella* 1,4,[5],12:i:- isolates were obtained from various sources, beef and pork products provided 84.6% of all tested isolates, proving to be the most important reservoirs in China.

The prevalence of MDR isolates observed in our surveillance of *Salmonella* Typhimurium (58.6%) and *Salmonella* 1,4,[5],12:i:- (76.9%) is similar to results from previous studies conducted in humans in China [2,21], and to rates of resistance reported in other countries [11,33]. The current study confirmed the prevalence of R-type ASSuT *Salmonella* Typhimurium isolates, with an even higher prevalence of this phenotype amongst *Salmonella* 1,4,[5],12:i:- isolates. In addition, several isolates displayed an R-type ACSSuT phenotype (Table 1). R-type ASSuT was also the most common profile among *Salmonella* 1,4,[5],12:i:- strains isolated in Germany from pigs, pork products, and humans [30], as well as among strains isolated from humans and foodstuffs in England, France, Germany, Italy, Spain, Switzerland, and the Netherlands [1,34]. The emergence and spread of the R-type ASSuT *Salmonella* 1,4,[5],12:i:- clonal lineage deserves further attention as it has been responsible for several foodborne *Salmonella* outbreaks, such as in Luxembourg in 2006 [18] and in Italy in 2010 [16], which can be associated with severe diseases, even deaths.

Most of the virulence genes with reported contributions to pathogenicity are located in *Salmonella* pathogenicity islands (SPIs), fimbrial clusters, plasmids and prophages. SPIs and fimbrial clusters are highly conserved regions, while prophages and plasmids are variable within the *Salmonella* genome. Some differences were observed in the genes located on prophages (*gipA*, *sodC1*, *sopE1*, *sspH1*) and plasmids (*spvC*, *pefA*, *rck*) when comparing virulence gene profiles from *Salmonella* Typhimurium and *Salmonella* 1,4,[5],12:i:- in previous studies [12,26]. In our study, three plasmid-borne virulence genes (*spvC*, *pefA*, and *rck*) were highly prevalent amongst *Salmonella* Typhimurium isolates, while none of the *Salmonella* 1,4,[5],12:i:- isolates contained these genes. This is consistent with the findings of Capuano et al [26]. The absence of these plasmid-borne virulence genes has been reported previously in R-type ASSuT *Salmonella* 1,4,[5],12:i:- strains of the European clone. In contrast, these genes have been identified in some Spanish clone *Salmonella* 1,4,[5],12:i:- strains, which are characterized by plasmid-mediated resistance to up to seven antimicrobial drugs, including ampicillin, chloramphenicol, gentamicin, streptomycin, sulfonamides, tetracyclines, and trimethoprim (R-type ACGSSuTTp) [12]. In addition, Capuano et al [26] also detected *gipA* in all *Salmonella* 1,4,[5],12:i:- isolates of food origin, but in only 76.9% of *Salmonell*a Typhimurium isolates. Another aspect to consider is that the detection of the target virulence genes showed no relationship to the serotype (*Salmonella* Typhimurium or *Salmonella* 1,4,[5],12:i:-), while some correlation with the MLST pattern was observed. All ST34 *Salmonell*a Typhimurium isolates, and a large number (76.9%) of the ST34 *Salmonella* 1,4,[5],12:i:- isolates, exhibited a virulence gene profile of “*gipA*; *sodC1*”.

All *Salmonella* 1,4,[5],12:i:- isolates in the present study were assigned to ST34, sharing identical STs with *Salmonella* Typhimurium isolates, which further confirmed that *Salmonella* 1,4,[5],12:i:- and *Salmonell*a Typhimurium were genetically closely related. Furthermore, these data suggested that *Salmonella* Typhimurium strains of ST34 might be ancestral candidates for serotype 1,4,[5],12:i:- from China. ST34 has previously been associated with R-type ASSuT *Salmonella* 1,4,[5],12:i:- strains belonging to the European clone [15,35]. In contrast, R-type ACGSSuTTp *Salmonella* 1,4,[5],12:i:- strains of the Spanish clone have been assigned to ST19 [12,35]. In the current study, *Salmonella* 1,4,[5],12:i:- isolates represented only one ST, while *Salmonella* Typhimurium isolates belonged to five different STs. This indicated considerably lower sequence diversity amongst *Salmonella* 1,4,[5],12:i:- than *Salmonella* Typhimurium. This observation has also been reported by Guerra et al [36] and Soyer et al [10].

All *Salmonella* 1,4,[5],12:i:- isolates examined in the current study exhibited deletions in the *fliBA* operon and flanking genes, although variations were identified. In previous studies, various deletion profiles affecting the *fljBA* operon and surrounding genes have been observed in *Salmonella* 1,4,[5],12:i:- strains. Most *Salmonella* 1,4,[5],12:i:- isolates from Spain appear to be characterized by deletions of *fljA*, *fljB*, *hin*, and *iroB* and conservation of STM2757 [12,13,28], whereas some of the American *Salmonella* 1,4,[5],12:i:- isolates seem to contain deletions that eliminate *fljA*, *fljB*, and STM2757 but maintain *hin* and *iroB* [7,10]. The deletion profile of *Salmonella* 1,4,[5],12:i:- strains from Italy is characterized by the absence of the *fljA*, *fljB*, and *hin* genes and the presence of STM2757, as in Spanish strains, but a conserved *iroB*, as with the American strains [14]. Among the isolates described in this work, *hin* and *iroB* were always conserved. The *ΔfljAB*-II deletion profile was identical to the American *Salmonella* 1,4,[5],12:i:- strains, whereas the *ΔfljAB*-I and *ΔfljAB*-III profiles were newly described for *Salmonella* 1,4,[5],12:i:- strains. This observed variation supported the independent evolution of multiple successful monophasic clones from *Salmonella* Typhimurium ancestors [5]. Sequencing of the entire *fljBA* operon and flanking genes in future studies could give better insight into the genetic background of such variants.

## Conclusions

This study is the first report of *Salmonella* 1,4,[5],12:i:- in food products from China. The main sources of *Salmonella* 1,4,[5],12:i:- in China appear to be pork and beef. The examined *Salmonella* 1,4,[5],12:i:- strains were mainly ST34, confirming the domination of the tetra-resistant ASSuT clone. The observed genomic deletion of the *fljBA* operon and its flanking genes in *Salmonella* 1,4,[5],12:i:- isolates revealed that multiple clones of this serovar are circulating in China, and several new clones were identified. Further studies are needed to assess these *Salmonella* 1,4,[5],12:i:- clones in more detail to obtain a better picture of the genetic background and relationship with *Salmonella* Typhimurium clones circulating in China.

## Supporting Information

S1 Fig Map of China illustrating provinces and cities included in the study. (TIF)

S2 Fig Identification of 13 monophasic *Salmonella* 1,4,[5],12:i:- and 58 biphasic *Salmonella* Typhimurium isolates by duplex-PCR. The isolates produced a single 1,000-bp amplicon corresponding to a fragment of the IS200 element were assigned to *Salmonella* 1,4,[5],12:i:- (lanes 35–39, 46, 51–53, 55, 58, 59, 62). The isolates produced two amplicons (1,000-bp and 1,389-bp) (lanes 2–23, 26–28, 30–34, 40–45, 47, 50, 54, 56, 57, 60, 63, 64, 66–70, 73–74, 76–79) or a single 1,389-bp amplicon (lanes 29, 61, 75) were assigned to *Salmonella* Typhimurium. M, DL2,000 DNA Marker. Lanes 1, 25, 49, 65, 72, *Salmonella* Typhimurium ATCC14028 reference strain; lanes 24, 48, 71, 80, water-only control.
(TIF)

S3 Fig Genomic deletion analysis of cluster V by PCR using two reference strains and 13 *Salmonella* 1,4,[5],12:i:- isolates. M, DL2,000 DNA Marker. C, water-only control. Lane 1, *Salmonella* Typhimurium ATCC14028 reference strain; lane 2, *Salmonella* Typhimurium CMCC50115 reference strain; lane 3, isolate 2011–33; lane 4, isolate 2011–34; lane 5, isolate 2012–35; lane 6, isolate 2012–36; lane 7, isolate 2012–37; lane 8, isolate 2013–45; lane 9, isolate 2013–50; lane 10, isolate 2013–51; lane 11, isolate 2013–52; lane 12, isolate 2014–54; lane 13, isolate 2013–57; lane 14, isolate 2013–58; lane 15, isolate 2013–62. a) *fljB*, DNA fragments of the expected sizes for *fljB* (lanes 1 and 2), no amplicon (lanes 3–15); b) *hin*, DNA fragments of the expected sizes for *hin* (lanes 1–15); c) *fljA*, DNA fragments of the expected sizes for *fljB* (lanes 1, 2, and 10), no amplicon (lanes 3–9, 11–15); d) *iroB*, DNA fragments of the expected sizes for *iroB* (lanes 1–15); e) STM2757, DNA fragments of the expected sizes for STM2757 (lanes 1–4, 6, 7, 9–10), no amplicon (lanes 5, 8, 12–15); f) STM2758, DNA fragments of the expected sizes for STM2758 (lanes 1–4, 6, 7, 9–10), no amplicon (lanes 5, 8, 12–15).
(TIF)

S1 Table Results of serotyping, antimicrobial resistance, virulotyping and MLST analysis of *Salmonella* isolates in this study. ^a^For antimicrobial abbreviation, ampicillin (AMP), amoxicillin-clavulanic acid (AMC), cephalothin (KF), cefazolin (KZ), cefoxitin (FOX), ceftriaxone (CRO), cefotaxime (CTX), ceftazidime (CAZ), cefoperazone (CFP), cefepime (FEP), chloramphenicol (C), tetracycline (TE), nalidixic acid (NA), ciprofloxacin (CIP), amikacin (AK), gentamicin (CN), streptomycin (S), kanamycin (K), trimethoprim-sulfamethoxazole (SXT), and sulfonamides (Su). Names of antimicrobials with capital letters means resistance; Names of antimicrobials with lowercase letters mean intermediate resistance; [tetra], a tetra-resistant pattern including resistance to ampicillin, streptomycin, sulfonamide, and tetracycline (ASSuT R-type); [penta], a penta-resistant pattern including resistance to ampicillin, chloramphenicol, streptomycin, sulfonamide, and tetracycline (ACSSuT R-type). ^b^VP1, virulence gene profile: *gipA*; *sodC1*; *spvC*; *pefA*; *rck*.
(DOC)

S2 Table Antimicrobial resistance profiles of *Salmonella* 1,4,[5],12:i:- isolates examined in this study. (DOC)

S3 Table Antimicrobial resistance profiles of *Salmonella* Typhimurium isolates examined in this study. (DOC)

## References

[1] Gallati C, Stephan R, Hächler H, Malorny B, Schroeter A, Nüesch-Inderbinen M. Characterization of *Salmonella enterica* subsp. *enterica* serovar 4,[5],12:i:- clones isolated from human and other sources in Switzerland between 2007 and 2011. Foodborne Pathog Dis. 2013;10: 549–554. 10.1089/fpd.2012.1407 23614800

[2] Deng X, Ran L, Wu S, Ke B, He D, Yang X, et al Laboratory-based surveillance of non-typhoidal Salmonella infections in Guangdong Province, China. Foodborne Pathog Dis. 2012;9: 305–312. 10.1089/fpd.2011.1008 22356574

[3] Majowicz SE, Musto J, Scallan E, Angulo FJ, Kirk M, O'Brien SJ, et al The global burden of nontyphoidal *Salmonella* gastroenteritis. Clin Infect Dis. 2010; 50: 882–889. 10.1086/650733 20158401

[4] European Food Safety Authority [EFSA], 2010 Scientific Opinion on monitoring and assessment of the public health risk of “*Salmonella* Typhimurium-like” strains. EFSA Journal. 8, 1826.

[5] Switt AI, Soyer Y, Warnick LD, Wiedmann M. Emergence, distribution, and molecular and phenotypic characteristics of *Salmonella enterica* serotype 4,5,12:i:-. Foodborne Pathog Dis. 2009;6: 407–415. 10.1089/fpd.2008.0213 19292687 PMC3186709

[6] Echeita MA Herrera S, Usera MA. Atypical, *fljB*-negative *Salmonella enterica* subsp. e*nterica* strain of serovar 4,5,12:i:- appears to be a monophasic variant of serovar Typhimurium. J Clin Microbiol. 2001;39: 2981–2983. 11474028 10.1128/JCM.39.8.2981-2983.2001 PMC88275

[7] Zamperini K, Soni V, Waltman D, Sanchez S, Theriault EC, Bray J, et al Molecular characterization reveals *Salmonella enterica* serovar 4,[5],12:i:- from poultry is a variant Typhimurium serovar. Avian Dis. 2007;51: 958–964. 18251408 10.1637/7944-021507-REGR.1

[8] Amavisit P, Boonyawiwat W, Bangtrakulnont A. Characterization of *Salmonella enterica* serovar Typhimurium and monophasic *Salmonella* serovar 1,4,[5],12:i:- isolates in Thailand. J Clin Microbiol. 2005;43: 2736–2740. 15956391 10.1128/JCM.43.6.2736-2740.2005 PMC1151962

[9] Kurosawa A, Imamura T, Tanaka K, Tamamura Y, Uchida I, Kobayashi A, et al Molecular typing of *Salmonella enterica* serotype Typhimurium and serotype 4,5,12:i:- isolates from cattle by multiple-locus variable-number tandem-repeats analysis. Vet Microbiol. 2012;160: 264–268. 10.1016/j.vetmic.2012.05.023 22717391

[10] Soyer Y, Switt AM, Davis MA, Maurer J, McDonough PL, Schoonmaker-Bopp DJ, et al *Salmonella enterica* serotype 4,5,12:i:-, an emerging *Salmonella* serotype that represents multiple distinct clones. J Clin Microbiol. 2009;47: 3546–3556. 10.1128/JCM.00546-09 19741087 PMC2772592

[11] Wasyl D, Hoszowski A. Occurrence and characterization of monophasic *Salmonella enterica* serovar typhimurium (1,4,[5],12:i:-) of non-human origin in Poland. Foodborne Pathog Dis. 2012;9: 1037–1043. 10.1089/fpd.2012.1154 23009171

[12] García P, Malorny B, Hauser E, Mendoza MC, Rodicio MR. Genetic Types, gene repertoire, and evolution of isolates of the *Salmonella enterica* serovar 4,5,12:i:- spanish clone assigned to different phage types. J Clin Microbiol. 2013;51: 973–978. 10.1128/JCM.02777-12 23325816 PMC3592032

[13] Laorden L, Herrera-León S, Martínez I, Sánchez A, Kromidas L, Bikandi J, et al Genetic evolution of the Spanish multidrug-resistant *Salmonella enterica* 4,5,12:i:- monophasic variant. J Clin Microbiol. 2010;48: 4563–4566. 10.1128/JCM.00337-10 20943866 PMC3008487

[14] Lucarelli C, Dionisi AM, Filetici E, Owczarek S, Luzzi I, Villa L. Nucleotide sequence of the chromosomal region conferring multidrug resistance (R-type ASSuT) in *Salmonella* Typhimurium and monophasic *Salmonella* Typhimurium strains. J Antimicrob Chemoth. 2012; 67: 111–114. 10.1093/jac/dkr391 21990047

[15] Hopkins KL, Kirchner M, Guerra B, Granier SA, Lucarelli C, Porrero MC, et al Multiresistant *Salmonella enterica* serovar 4,[5],12:i:- in Europe: a new pandemic strain? Eurosurveillance. 2010a;15: 19580 20546690

[16] Barco L, Ramon E, Cortini E, Longo A, Dalla Pozza MC, Lettini AA, et al Molecular characterization of *Salmonella enterica* serovar 4,[5],12:i:- DT193 ASSuT strains from two outbreaks in Italy. Foodborne Pathog Dis. 2014;11: 138–144. 10.1089/fpd.2013.1626 24328499

[17] Bone A, Noel H, Le Hello S, Pihier N, Danan C, Raguenaud ME, et al Nationwide outbreak of *Salmonella enterica* serotype 4,12:i:- infections in France, linked to dried pork sausage. Eurosurveillance. 2010;15: 19592 20576238

[18] Mossong J, Marques P, Ragimbeau C, Huberty-Krau P, Losch S, Meyer G, et al Outbreaks of monophasic *Salmonella enterica* serovar 4,[5],12:i:- in Luxembourg, 2006. Eurosurveillance. 2007;12: 719. 10.2807/esm.12.06.00719-en 17991400

[19] Centers for Disease Control and Prevention [CDC]. The emergence and antimicrobial resistance of *Salmonella* serotype I 4,[5],12:i:- in the United States, 2006 Atlanta, GA: CDC.

[20] Mulvey MR, Finley R, Allen V, Ang L, Bekal S, El Bailey S, et al Emergence of multidrug-resistant *Salmonella enterica* serotype 4,[5],12:i:- involving human cases in Canada: results from the Canadian Integrated Program on Antimicrobial Resistance Surveillance (CIPARS), 2003–10. J Antimicrob Chemoth. 2013; 68: 1982–1986. 10.1093/jac/dkt149 23710071

[21] Liang ZM, Ke BX, Deng XL, He DM, Ke CW, Li BS, et al Characteristics of main human-source *Salmonella* serovars resistant to quinolone in Guangdong Province, 2010–2011. South China Journal of Preventive Medicine. 2013;39: 27–32.

[22] Grimont PAD and Weill FX, 2007 Antigenic Formulae Fort the *Salmonella* Serovars, 9th edition Paris, France: WHO Collaborating Centre for Reference and Research on Salmonella.

[23] Chiou CS, Huang JF, Tsai LH, Hsu KM, Liao CS, Chang HL. A simple and low-cost paper-bridged method for *Salmonella* phase reversal. Diagn Micr Infec Dis. 2006; 54: 315–317. 10.1016/j.diagmicrobio.2005.10.009 16466895

[24] Tennant SM, Diallo S, Levy H, Livio S, Sow SO, Tapia M, et al Identification by PCR of non-typhoidal *Salmonella enterica* serovars associated with invasive infections among febrile patients in Mali. PLoS Neglect Trop D. 2010;4: e621. 10.1371/journal.pntd.0000621 PMC2834738 20231882

[25] Clinical and Laboratory Standards Institute. Performance Standards for Antimicrobial Susceptibility Testing; 24th informational supplement CLSI documents M100-S24. CLSI, 2014.

[26] Capuano F, Mancusi A, Capparelli R, Esposito S, Proroga YT. Characterization of drug resistance and virulotypes of *Salmonella* strains isolated from food and humans. Foodborne Pathog Dis. 2013;10: 963–968. 10.1089/fpd.2013.1511 24102078

[27] Huehn S, La Ragione RM, Anjum M, Saunders M, Woodward MJ, Bunge C, et al Virulotyping and antimicrobial resistance typing of *Salmonella enterica* serovars relevant to human health in Europe. Foodborne Pathog Dis. 2010;7: 523–535. 10.1089/fpd.2009.0447 20039795

[28] Garaizar J, Porwollik S, Echeita A, Rementeria A, Herrera S, Wong RM, et al DNA microarray-based typing of an atypical monophasic *Salmonella enterica* serovar. J Clin Microbiol. 2002;40: 2074–2078. 12037067 10.1128/JCM.40.6.2074-2078.2002 PMC130817

[29] Boland C, Bertrand S, Mattheus W, Dierick K, Wattiau P. Molecular typing of monophasic *Salmonella* 4,[5]:i:- strains isolated in Belgium (2008–2011). Vet Microbiol. 2014;168: 447–450. 10.1016/j.vetmic.2013.11.040 24398228

[30] Hauser E, Tietze E, Helmuth R, Junker E, Blank K, Prager R, et al Pork contaminated with *Salmonella enterica* serovar 4,[5],12:i:-, an emerging health risk for humans. Appl Environ Microb. 2010;76: 4601–4610. 10.1128/AEM.02991-09 PMC2901716 20472721

[31] de la Torre E, Zapata D, Tello M, Mejía W, Frías N, García Peña FJ, et al Several *Salmonella enterica* subsp. *enterica* serotype 4,5,12:i:- phage types isolates from swine samples originate from serotype Typhimurium DT U302. J Clin Microbiol. 2003;41: 2395–2400. 12791855 10.1128/JCM.41.6.2395-2400.2003 PMC156524

[32] Barco L, Mancin M, Ruffa M, Saccardin C, Minorello C, Zavagnin P, et al Application of the Random Forest method to analyse epidemiological and phenotypic characteristics of *Salmonella* 4,[5],12:i:- and *Salmonella* Typhimurium strains. Zoonoses Public Hlth. 2012;59: 505–512. 10.1111/j.1863-2378.2012.01487.x 22583909

[33] Dionisi AM, Graziani C, Lucarelli C, Filetici E, Villa L, Owczarek S, et al Molecular characterization of multidrug-resistant strains of *Salmonella enterica* serotype Typhimurium and monophasic variant (S. 4,[5],12:i:-) isolated from human infections in Italy. Foodborne Pathog Dis. 2009;6: 711–717. 10.1089/fpd.2008.0240 19580448

[34] Hopkins KL, Nair S, Kirchner M, Guerra B, Granier S, Lucarelli C, et al Genetic variation in emerging multidrug-resistant *Salmonella enterica* 4,[5],12:i:- from seven European countries, S1:1, p 13. Abstr. 2nd Antimicrob. Resist. Zoonotic Bacteria Foodborne Pathog Animals Hum. Environ. Meet. Am. Soc. Microbiol. American Society for Microbiology, Washington, DC, 2010b.

[35] Antunes P, Mourao J, Pestana N, Peixe L. Leakage of emerging clinically relevant multidrug-resistant *Salmonella* clones from pig farms. J Antimicrob Chemoth. 2011;66: 2028–2032. 10.1093/jac/dkr228 21697179

[36] Guerra B, Laconcha I, Soto SM, Gonzalez-Hevia MA, Mendoza MC. Molecular characterisation of emergent multiresistant *Salmonella enterica* serotype [4,5,12:I:-] organisms causing human salmonellosis. Fems Microbiol Lett. 2000; 190: 341–347. 11034302 10.1111/j.1574-6968.2000.tb09309.x

