# Supplementary figures and images for: Prevalence and Characterization of Monophasic Salmonella Serovar 1,4,[5],12:i:- of Food Origin in China
Source: PLoS One. 2015 Sep 11;10(9):e0137967. doi: 10.1371/journal.pone.0137967 (PMC4567320; doi:10.1371/journal.pone.0137967)

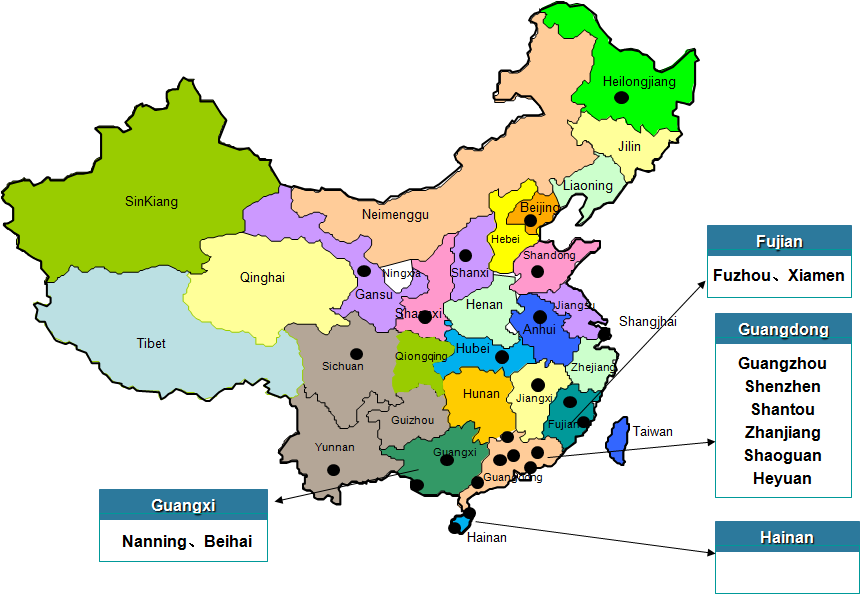

Supplement: S1 Fig — (TIF) [file pone.0137967.s001.tif]

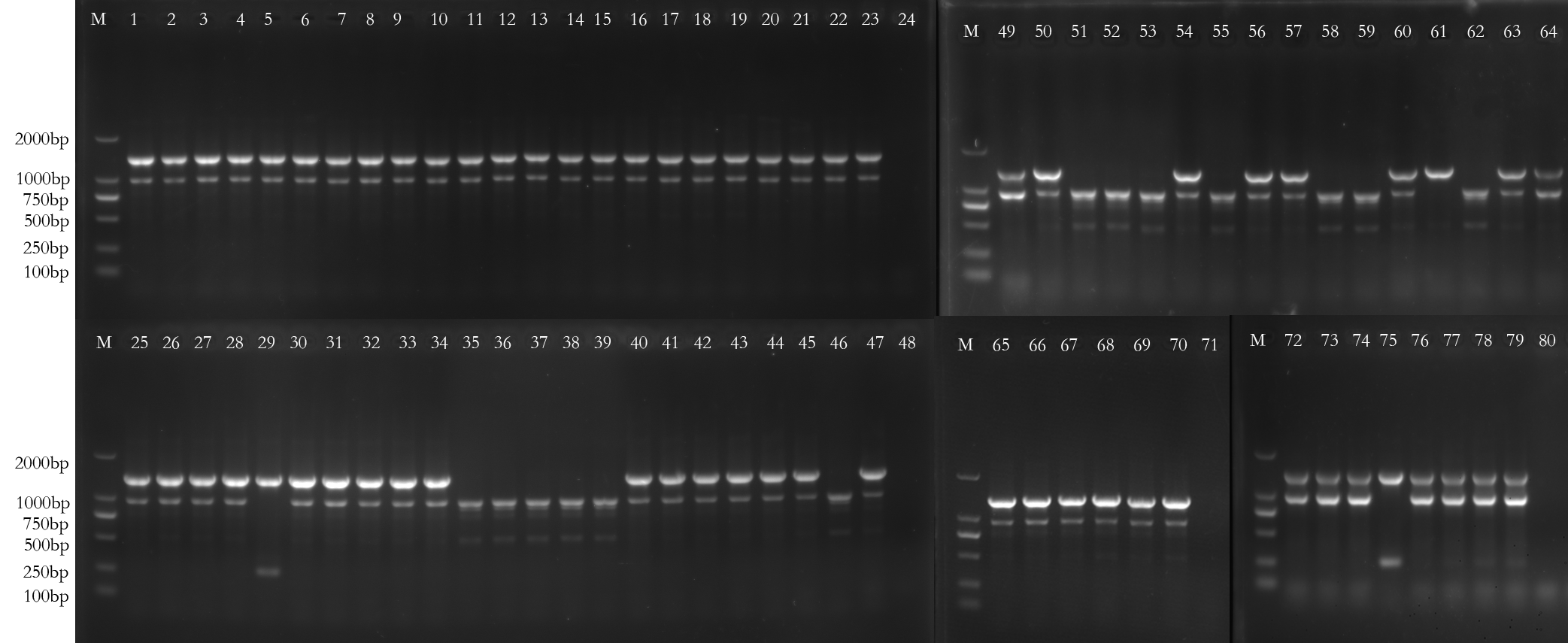

Supplement: S2 Fig — The isolates produced a single 1,000-bp amplicon corresponding to a fragment of the IS200 element were assigned to Salmonella 1,4,[5],12:i:- (lanes 35–39, 46, 51–53, 55, 58, 59, 62). The isolates produced two amplicons (1,000-bp and 1,389-bp) (lanes 2–23, 26–28, 30–34, 40–45, 47, 50, 54, 56, 57, 60, 63, 64, 66–70, 73–74, 76–79) or a single 1,389-bp amplicon (lanes 29, 61, 75) were assigned to Salmonella Typhimurium. M, DL2,000 DNA Marker. Lanes 1, 25, 49, 65, 72, Salmonella Typhimurium ATCC14028 reference strain; lanes 24, 48, 71, 80, water-only control. (TIF) [file pone.0137967.s002.tif]

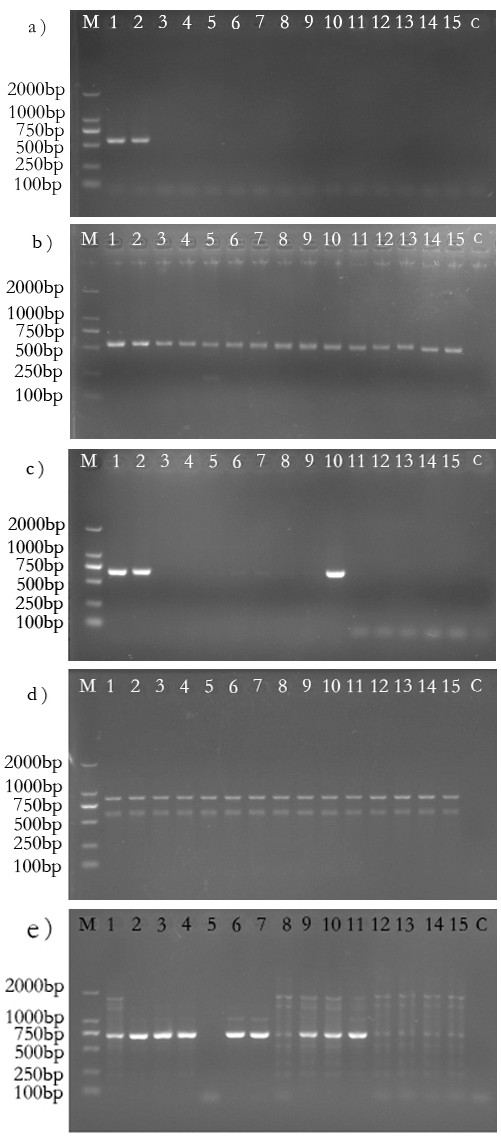

Supplement: S3 Fig — M, DL2,000 DNA Marker. C, water-only control. Lane 1, Salmonella Typhimurium ATCC14028 reference strain; lane 2, Salmonella Typhimurium CMCC50115 reference strain; lane 3, isolate 2011–33; lane 4, isolate 2011–34; lane 5, isolate 2012–35; lane 6, isolate 2012–36; lane 7, isolate 2012–37; lane 8, isolate 2013–45; lane 9, isolate 2013–50; lane 10, isolate 2013–51; lane 11, isolate 2013–52; lane 12, isolate 2014–54; lane 13, isolate 2013–57; lane 14, isolate 2013–58; lane 15, isolate 2013–62. a) fljB, DNA fragments of the expected sizes for fljB (lanes 1 and 2), no amplicon (lanes 3–15); b) hin, DNA fragments of the expected sizes for hin (lanes 1–15); c) fljA, DNA fragments of the expected sizes for fljB (lanes 1, 2, and 10), no amplicon (lanes 3–9, 11–15); d) iroB, DNA fragments of the expected sizes for iroB (lanes 1–15); e) STM2757, DNA fragments of the expected sizes for STM2757 (lanes 1–4, 6, 7, 9–10), no amplicon (lanes 5, 8, 12–15); f) STM2758, DNA fragments of the expected sizes for STM2758 (lanes 1–4, 6, 7, 9–10), no amplicon (lanes 5, 8, 12–15). (TIF) [file pone.0137967.s003.tif]
