# Supplementary material for: Prevalence and Characterization of Monophasic Salmonella Serovar 1,4,[5],12:i:- of Food Origin in China
Source: PLoS One. 2015 Sep 11;10(9):e0137967. doi: 10.1371/journal.pone.0137967 (PMC4567320; doi:10.1371/journal.pone.0137967)
Supplement: S1 Table — aFor antimicrobial abbreviation, ampicillin (AMP), amoxicillin-clavulanic acid (AMC), cephalothin (KF), cefazolin (KZ), cefoxitin (FOX), ceftriaxone (CRO), cefotaxime (CTX), ceftazidime (CAZ), cefoperazone (CFP), cefepime (FEP), chloramphenicol (C), tetracycline (TE), nalidixic acid (NA), ciprofloxacin (CIP), amikacin (AK), gentamicin (CN), streptomycin (S), kanamycin (K), trimethoprim-sulfamethoxazole (SXT), and sulfonamides (Su). Names of antimicrobials with capital letters means resistance; Names of antimicrobials with lowercase letters mean intermediate resistance; [tetra], a tetra-resistant pattern including resistance to ampicillin, streptomycin, sulfonamide, and tetracycline (ASSuT R-type); [penta], a penta-resistant pattern including resistance to ampicillin, chloramphenicol, streptomycin, sulfonamide, and tetracycline (ACSSuT R-type). bVP1, virulence gene profile: gipA; sodC1; spvC; pefA; rck. (DOC) [file pone.0137967.s004.doc]

**S1 Table.** **Results of serotyping, antimicrobial resistance, virulotyping and MLST analysis of *Salmonella* isolates in this study.**

| **Year-isolate**  **no.** | **Sample** | **Sample character** | **Sample city** | **Purchase location** | **Serotype** | **MLST pattern** | **Resistance profile ^a^** | **Virulence gene profile ^b^** |
| --- | --- | --- | --- | --- | --- | --- | --- | --- |
| 2011-1 | Pork | Fresh  minced meat | Guangzhou | Shaheding market | Typhimurium | ST34 | NA-AMP-TE-s-su | *gipA*; *sodC1* |
| 2011-2 | Pork | Fresh  meat slices | Shaoguan | Huifu chain market | Typhimurium | ST19 | [penta]-NA-CN-SXT-K | *gipA*; *sodC1* |
| 2011-3 | Pork | Fresh  meat slices | Shaoguan | Huifu chain market | Typhimurium | ST34 | [penta]-NA-cip-amc-cn-SXT-K | *gipA*; *sodC1* |
| 2011-4 | Duck | Fresh  meat slices | Zhanjiang | Old dongfeng market | Typhimurium | ST34 | AMP-C-TE-s-Su-NA-amc-CFP-cn-SXT-K | *gipA*; *sodC1* |
| 2012-6 | Duck | Fresh  meat slices | Haikou | Longshepo market | Typhimurium | ST19 | NA-amp-su | VP1 |
| 2012-7 | Duck | Fresh  meat slices | Beihai | Beijing road market | Typhimurium | ST19 | NA-s-su | VP1 |
| 2012-8 | Chicken | Frozen  broiler wing | Beihai | Guizhou road market | Typhimurium | ST34 | [tetra]-NA-sxt | *gipA*; *sodC1* |
| 2012-9 | Goose | Fresh  meat slices | Beihai | Changqing market | Typhimurium | ST19 | AMP-C-TE-s-Su-CAZ | *sodC1* |
| 2012-10 | Pork | Fresh  minced meat | Nanning | Nancheng supermarket | Typhimurium | ST19 | AMP-C-TE-s-Su-NA-CIP-AMC | VP1 |
| 2012-11 | Duck | Fresh  meat slices | Nanning | Dancun market | Typhimurium | ST19 | AMP-TE-S-su-NA-amc | VP1 |
| 2012-12 | Chicken | Frozen  broiler wing | Nanning | Luban market | Typhimurium | ST19 | NA-s-su | VP1 |
| 2012-13 | Pork | Fresh  minced meat | Fuzhou | Yonghui supermarket | Typhimurium | ST19 | NA-AMP | VP1 |
| 2012-14 | Duck | Fresh  meat slices | Fuzhou | Yonghui supermarket | Typhimurium | ST19 | [tetra]-NA-CIP-AMC-KF-FOX-CN-K | VP1 |
| 2012-15 | Pork | Frozen  minced meat | Xiamen | Walmart | Typhimurium | ST19 | [penta]-NA-ak-AMC-KF-CTX-KZ-cfp-FOX-CN-SXT-K | *gipA*; *sodC1* |
| 2012-16 | Chicken | Fresh  meat slices | Xiamen | The eight market | Typhimurium | ST19 | NA-CTX | VP1 |
| 2012-17 | Chicken | Frozen  broiler leg | Nanchang | Dunzitang fair | Typhimurium | ST19 | Su-NA-amp | VP1 |
| 2012-18 | Pork | Pork tongue | Shanghai | Pusan road market | Typhimurium | ST19 | NA | VP1 |
| 2012-19 | Oyster | Husked in water | Shanghai | Hengda aquatic wholesale market | Typhimurium | ST19 | AMP-TE-s-na-cip-AMC-sxt | *sodC1* |
| 2012-20 | Fish | Chilled | Shanghai | Pusan road market | Typhimurium | ST19 | AMP-c-TE-s-na-CIP-AMC-sxt | *sodC1* |
| 2012-21 | Fish | Live | Shanghai | Pusan road market | Typhimurium | ST19 | AMP-TE-na-cip-AMC-CRO-sxt | *sodC1* |
| 2012-22 | Chicken | Frozen  broiler wing | Shanghai | Pusan road market | Typhimurium | ST34 | [tetra]-NA-cip-amc-SXT-K | *gipA*; *sodC1* |
| 2012-23 | Mutton | Frozen  meat slices | Shanghai | Shanghai Yunlian market | Typhimurium | ST19 | NA-s | VP1 |
| 2012-24 | Chicken | Frozen  broiler leg | Nanchang | Dunzitang fair | Typhimurium | ST19 | NA-su | VP1 |
| 2012-25 | Chicken | Frozen  broiler leg | Nanchang | Dunzitang fair | Typhimurium | ST19 | S-Su-NA | VP1 |
| 2012-26 | Pork | Frozen  minced meat | Nanchang | Walmart | Typhimurium | ST19 | C-TE-s-Su-NA-cn-SXT-K | *sodC1* |
| 2012-28 | Chicken | Frozen  broiler wing | Nanchang | Xi'mazhuang fair | Typhimurium | ST19 | NA-s-su | VP1 |
| 2012-29 | Chicken | Fresh  meat slices | Chengdu | Xiaojiahe market | Typhimurium | ST34 | AMP-C-TE-s-Su-NA-cip-amc-cn-SXT-K | *gipA*; *sodC1* |
| 2012-30 | Pork | Fresh  meat slices | Chengdu | Yankou people's shopping malls | Typhimurium | ST19 | AMP-C-s-Su-SXT | *sodC1* |
| 2012-31 | Chicken | Fresh  meat slices | Kunming | Tianyuanli yintan market | Typhimurium | ST19 | NA-amp-s-su | VP1 |
| 2012-32 | Chicken | Fresh  meat slices | Taiyuan | Dongan road comprehensive trade market | Typhimurium | ST19 | amp | VP1 |
| 2011-38 | Duck | Fresh  meat slices | Shenzhen | Yantian wholesale market | Typhimurium | ST19 | CIP-KZ-SXT | *sodC1*; *spvC*; *pefA*; *rck* |
| 2011-39 | Duck | Fresh  meat slices | Zhanjiang | Old dongfeng market | Typhimurium | ST34 | AMP-C-TE-s-Su-NA-cip-amc-cn-SXT-K | *gipA*; *sodC1* |
| 2013-41 | Pigeon | Live | Shenzhen | Hubei east market | Typhimurium | ST19 | TE-S-Su-NA | VP1 |
| 2013-42 | Pigeon | Live | Shenzhen | Hubei east market | Typhimurium | ST19 | TE-S-Su-NA-k | VP1 |
| 2013-43 | Chicken | Frozen  broiler leg | Lanzhou | China Resources Vanguard | Typhimurium | ST19 | [penta]-NA-CIP-amc-CN-SXT-k | VP1 |
| 2013-44 | Mutton | Fresh  meat slices | Lanzhou | Muta road market | Typhimurium | ST19 | Su-NA | VP1 |
| 2013-47 | Chicken | Fresh meat slices | Guangzhou | Xiajie fair | Typhimurium | ST19 | AMP-TE-S-NA | VP1 |
| 2013-49 | Chicken | Fresh meat slices | Shantou | Donghu street market | Typhimurium | ST19 | [tetra]-NA-amc-KZ-K | VP1 |
| 2014-53 | Pork | Fresh meat slices | Kunming | Carrefour | Typhimurium | ST19 | [penta]-amc-CFP-SXT-k | *sodC1* |
| 2013-55 | Duck | Frozen duck leg | Haerbin | Walmart | Typhimurium | ST19 | NA-KZ | VP1 |
| 2013-56 | Duck | Frozen duck leg | Haerbin | Walmart | Typhimurium | ST19 | AMP-s-su-NA-amc-KF-CRO-CTX-CAZ-KZ-FEP-CFP | VP1 |
| 2013-60 | Chicken | Fresh meat slices | Xiamen | Ruijing market | Typhimurium | ST19 | s-su-NA-SXT | VP1 |
| 2013-63 | Duck | Fresh meat slices | Beihai | Guizhou road market | Typhimurium | ST34 | [penta]-NA-cip-amc-cn-SXT-k | *gipA*; *sodC1* |
| 2014-66 | Pork | Fresh meat slices | Sanya | Wanghao supermarket | Typhimurium | ST19 | [tetra]-NA-amc-kf-CN-SXT-K | VP1 |
| 2014-67 | Pigeon | Live | Sanya | The one market | Typhimurium | ST1922 | s-su-CIP | VP1 |
| 2014-68 | Chicken | Fresh meat slices | Sanya | City center market | Typhimurium | ST19 | s-Su-NA-kz | VP1 |
| 2014-69 | Duck | Fresh meat slices | Sanya | The one market | Typhimurium | ST19 | s-su-NA-KZ | VP1 |
| 2013-70 | Chicken | Frozen meat slices | Haikou | Longshepo market | Typhimurium | ST19 | s-su-NA | VP1 |
| 2013-71 | Duck | Fresh meat slices | Haikou | Longshepo market | Typhimurium | ST19 | Su-NA-amc | *sodC1*; *spvC*; *pefA*; *rck* |
| 2013-74 | Fish | Live | Guangzhou | Yonghenglong west street supermarket | Typhimurium | ST19 | TE-S-NA | VP1 |
| 2013-75 | Fish | Live | Guangzhou | Yonghenglong west street supermarket | Typhimurium | ST19 | TE-S-NA | VP1 |
| 2013-77 | Fish | Live | Shenzhen | Hubei east market | Typhimurium | ST34 | AMP-C-s-Su-NA-cip-amc-cn-SXT-K | *gipA*; *sodC1* |
| 2013-78 | Shrimp | Live | Shenzhen | Hubei east market | Typhimurium | ST19 | s-NA | VP1 |
| 2013-79 | Fish | Live | Xian | Tanshi street market | Typhimurium | ST34 | [penta]-NA-KZ-cn-SXT-K | *gipA*; *sodC1* |
| 2013-80 | Fish | Live | Beijing | Lottemart | Typhimurium | ST19 | AMP-C-TE-na-AMC-sxt | VP1 |
| 2013-76 | Fish | Chilled | Shenzhen | China Resources Vanguard | Typhimurium | ST36 | s-na | *sodC1*; *sopE1* |
| 2012-27 | Chicken | Fresh meat slices | Nanchang | Xi'mazhuang fair | Typhimurium | ST1544 | Su-amp | VP1 |
| 2013-61 | Duck | Fresh meat slices | Xiamen | Tesco | Typhimurium | ST1544 | NA-su | VP1 |
| 2011-33 | Beef | Fresh meat slices | Guangzhou | Huangbian wholesale market | 1,4,[5],12:i:- | ST34 | [penta]-NA-CIP-amc-cn-SXT-k | *gipA*; *sodC1* |
| 2011-34 | Beef | Fresh meat slices | Guangzhou | Huangbian wholesale market | 1,4,[5],12:i:- | ST34 | AMP-C-TE-S-NA-CIP-amc-cn-SXT-k | *gipA*; *sodC1* |
| 2012-35 | Beef | Fresh meat slices | Beihai | RT-MART | 1,4,[5],12:i:- | ST34 | [tetra]-na-K | *gipA*; *sodC1* |
| 2012-36 | Beef | Fresh meat slices | Taiyuan | Upwell supermarket | 1,4,[5],12:i:- | ST34 | AMP-TE-Su-NA-CRO-cfp-CN | *gipA*; *sodC1* |
| 2012-37 | Beef | Fresh meat slices | Taiyuan | Upwell supermarket | 1,4,[5],12:i:- | ST34 | [tetra]-NA-K | *gipA*; *sodC1* |
| 2013-45 | Pigeon | Live | Lanzhou | Muta road market | 1,4,[5],12:i:- | ST34 | TE-s-su-k | *gipA*; *sodC1*; *sopE1* |
| 2013-50 | Pork | Fresh meat slices | Heyuan | Guangsheng market | 1,4,[5],12:i:- | ST34 | AMP-C-TE-s-Su-NA-cip-AMC-CN-K | *gipA*; *sodC1* |
| 2013-51 | Beef | Fresh meat slices | Heyuan | Renrenle supermarket | 1,4,[5],12:i:- | ST34 | [tetra]-NA-amc-kf-kz-k | *gipA*; *sodC1* |
| 2013-52 | Chicken | Fresh meat slices | Heyuan | Yuancheng central market | 1,4,[5],12:i:- | ST34 | AMP-C-TE-s-Su-NA-cip-amc-KZ-FOX-CN-K | *gipA*; *sodC1* |
| 2014-54 | Pork | Fresh  minced meat | Kunming | Carrefour | 1,4,[5],12:i:- | ST34 | [tetra]-na-amc-KZ-k | *gipA*; *sodC1* |
| 2013-57 | Pork | Fresh meat slices | Taiyuan | Wulongkou seafood market | 1,4,[5],12:i:- | ST34 | TE-s-su-cro | *gipA*; *sodC1*; *sopE1* |
| 2013-58 | Pork | Fresh meat slices | Taiyuan | Wulongkou seafood market | 1,4,[5],12:i:- | ST34 | TE-s-su-CFP | *gipA*; *sodC1*; *sopE1* |
| 2013-62 | Pork | Fresh  minced meat | Beihai | RT-Mart | 1,4,[5],12:i:- | ST34 | [penta]-NA-amc-cn-SXT-K | *gipA*; *sodC1* |

^a^For antimicrobial abbreviation, ampicillin (AMP), amoxicillin-clavulanic acid (AMC), cephalothin (KF), cefazolin (KZ), cefoxitin (FOX), ceftriaxone (CRO), cefotaxime (CTX), ceftazidime (CAZ), cefoperazone (CFP), cefepime (FEP), chloramphenicol (C), tetracycline (TE), nalidixic acid (NA), ciproﬂoxacin (CIP), amikacin (AK), gentamicin (CN), streptomycin (S), kanamycin (K), trimethoprim-sulfamethoxazole (SXT), and sulfonamides (Su). Name of antimicrobials with capital letters means resistance; Name of antimicrobials with lowercase letters means intermediate resistance; [tetra], a tetra-resistant pattern including resistance to ampicillin, streptomycin, sulfonamide, and tetracycline (ASSuT R-type); [penta], a penta-resistant pattern including resistance to ampicillin, chloramphenicol, streptomycin, sulfonamide, and tetracycline (ACSSuT R-type).

^b^VP1, virulence gene profile: *gipA*; *sodC1*; *spvC*; *pefA*; *rck*.

.
