# Supplementary material for: Prevalence and Characterization of Monophasic Salmonella Serovar 1,4,[5],12:i:- of Food Origin in China
Source: PLoS One. 2015 Sep 11;10(9):e0137967. doi: 10.1371/journal.pone.0137967 (PMC4567320; doi:10.1371/journal.pone.0137967)
Supplement: S2 Table — (DOC) [file pone.0137967.s005.doc]

**S2 Table. Antimicrobial resistance profiles of *Salmonella* 1,4,[5],12:i:- isolates examined in this study.**

| **Antimicrobial agents** | **No. of isolates (%)** | | |
| --- | --- | --- | --- |
|  | **Resistant (R)** | **Intermediate (I)** | **Susceptible (S)** |
| **β-Lactams** |  |  |  |
| **Ampicillin** | 10 (76.9) | 0 (0.0) | 3 (23.1) |
| **Amoxicillin-clavulanic acid** | 1 (7.7) | 6 (46.2) | 6 (46.2) |
| **Cephalothin** | 0 (0.0) | 1 (7.7) | 12 (92.3) |
| **Cefazolin** | 2 (15.4) | 1 (7.7) | 10 (76.9) |
| **Cefoxitin** | 1 (7.7) | 0 (0.0) | 12 (92.3) |
| **Ceftriaxone** | 1 (7.7) | 1 (7.7) | 11 (84.6) |
| **Cefotaxime** | 0 (0.0) | 0 (0.0) | 13 (100.0) |
| **Ceftazidime** | 0 (0.0) | 0 (0.0) | 13 (100.0) |
| **Cefoperazone** | 1 (7.7) | 1 (7.7) | 11 (84.6) |
| **Cefepime** | 0 (0.0) | 0 (0.0) | 13 (100.0) |
| **Phenicols** |  |  |  |
| **Chloramphenicol** | 5 (38.5) | 0 (0.0) | 8 (61.5) |
| **Tetracyclines** |  |  |  |
| **Tetracycline** | 13 (100.0) | 0 (0.0) | 0 (0.0) |
| **Quinolones and fluoroquinolones** |  |  |  |
| **Nalidixic acid** | 8 (61.5) | 2 (15.4) | 3 (23.1) |
| **Ciproflaxin** | 2 (15.4) | 2 (15.4) | 9 (69.2) |
| **Aminoglycosides** |  |  |  |
| **Amikacin** | 0 (0.0) | 0 (0.0) | 13 (100.0) |
| **Gentamicin** | 3 (23.1) | 3 (23.1) | 7 (53.8) |
| **Streptomycin** | 7 (53.8) | 5 (38.5) | 1 (7.7) |
| **Kanamycin** | 5 (38.5) | 5 (38.5) | 3 (23.1) |
| **Sulfonamides and synergistic agents** |  |  |  |
| **Trimethoprim-sulfamethoxazole** | 3 (23.1) | 0 (0.0) | 10 (76.9) |
| **Sulfonamides** | 10 (76.9) | 3 (23.1) | 0 (0.0) |
| **Pansusceptible** | 0 (0.0) |  |  |
| **≥1 Antimicrobial** | 13 (100.0) |  |  |
| **≥3 Antimicrobials** | 10 (76.9) |  |  |
