# Supplementary material for: Prevalence and Characterization of Monophasic Salmonella Serovar 1,4,[5],12:i:- of Food Origin in China
Source: PLoS One. 2015 Sep 11;10(9):e0137967. doi: 10.1371/journal.pone.0137967 (PMC4567320; doi:10.1371/journal.pone.0137967)
Supplement: S3 Table — (DOC) [file pone.0137967.s006.doc]

**S3 Table. Antimicrobial resistance profiles of *Salmonella*** Typhimurium isolates examined in this study.

| **Antimicrobial agents** | **No. of isolates (%)** | | |
| --- | --- | --- | --- |
|  | **Resistant (R)** | **Intermediate (I)** | **Susceptible (S)** |
| **β-Lactams** |  |  |  |
| **Ampicillin (10 μg)** | 28 (48.3) | 5 (8.6) | 25 (43.1) |
| **Amoxicillin-clavulanic acid (30 μg)** | 7 (12.1) | 14 (24.1) | 37 (63.8) |
| **Cephalothin (30 μg)** | 3 (5.2) | 1 (1.7) | 54 (93.1) |
| **Cefazolin (30 μg)** | 7 (12.1) | 1 (1.7) | 50 (86.2) |
| **Cefoxitin (30 μg)** | 2 (3.4) | 0 (0.0) | 56 (96.6) |
| **Ceftriaxone (30 μg)** | 2 (3.4) | 0 (0.0) | 56 (96.6) |
| **Cefotaxime (30 μg)** | 3 (5.2) | 0 (0.0) | 55 (94.8) |
| **Ceftazidime (30 μg)** | 2 (3.4) | 0 (0.0) | 56 (96.6) |
| **Cefoperazone (75 μg)** | 3 (5.2) | 1 (1.7) | 54 (93.1) |
| **Cefepime (30 μg)** | 1 (1.7) | 0 (0.0) | 57 (98.3) |
| **Phenicols** |  |  |  |
| **Chloramphenicol (30 μg)** | 16 (27.6) | 1 (1.7) | 41 (70.7) |
| **Tetracyclines** |  |  |  |
| **Tetracycline (30 μg)** | 29 (50.0) | 0 (0.0) | 29 (50.0) |
| **Quinolones and fluoroquinolones** |  |  |  |
| **Nalidixic acid (30 μg)** | 46 (79.3) | 5 (8.6) | 7 (12.1) |
| **Ciproflaxin (5 μg)** | 6 (10.3) | 8 (13.8) | 44 (75.9) |
| **Aminoglycosides** |  |  |  |
| **Amikacin (30 μg)** | 0 (0.0) | 1 (1.7) | 57 (98.3) |
| **Gentamicin (10 μg)** | 5 (8.6) | 8 (13.8) | 45 (77.6) |
| **Streptomycin (10 μg)** | 18 (31.0) | 25 (43.1) | 15 (25.9) |
| **Kanamycin (30 μg)** | 15 (25.9) | 6 (10.3) | 37 (63.8) |
| **Sulfonamides and synergistic agents** |  |  |  |
| **Trimethoprim-sulfamethoxazole (25 μg)** | 17 (29.3) | 1 (1.7) | 40 (69.0) |
| **Sulfonamides (300 μg)** | 36 (62.1) | 14 (24.1) | 8 (13.8) |
| **Pansusceptible** | 0 (0.0) |  |  |
| **≥1 Antimicrobial** | 56 (96.6) |  |  |
| **≥3 Antimicrobials** | 34 (58.6) |  |  |
